# Measles outbreaks and Supplemental Immunization Activities (SIAs): the Gwagwalada experience, Abuja 2015

**DOI:** 10.11604/pamj.supp.2019.32.1.13368

**Published:** 2019-01-24

**Authors:** Olukemi Titilope Olugbade, Adeniran Sunday Adeyemi, Adedotun Hadizah Adeoti, Olayinka Stephen Ilesanmi, Saheed Oluwatoyin Gidado, Ndadilnasiya Endie Waziri, Mabel Kamweli Aworh

**Affiliations:** 1 Nigeria Field Epidemiology and Laboratory Training Programme, Abuja, Nigeria; 2 Epidemiology and Surveillance Unit, Department of Primary Healthcare, Gwagwalada Area Council, Abuja, FCT, Nigeria; 3 Epidemiology Unit, Ministry of Health, Ibadan, Oyo State, Nigeria; 4 National Stop Transmission of Polio Programme (NSTOP), Abuja, Nigeria; 5 Department of Veterinary and Pest Services, Federal Ministry of Agriculture & Rural Development, Abuja, Nigeria

**Keywords:** Immunization, hard to reach, measles, outbreaks, surveillance, vaccination, Nigeria

## Abstract

**Introduction:**

In November 2015 a measles outbreak was detected in several clustered settlements during the Northern Measles Supplementary Immunization Activities (SIAs) campaign in Gwagwalada, Nigeria, a measles outbreak was detected. Six weeks later another outbreak with 17 cases was reported in a different settlement in the same area council in December 2015 and January 2016. An outbreak investigation was initiated to characterize the outbreak in terms of time and person and implement prevention and control measures.

**Methods:**

Suspected cases were defined as any person in Gwagwalada with onset of fever and rash between 1st November 2015 and 12th January 2016. Probable cases were defined as suspected cases with 3 days of rash or known exposure to someone with laboratory-confirmed measles. Confirmed case patients were defined as suspected or probable cases with Koplik spots or positive titer for immunoglobulin (Ig) M antibody. We conducted house to house case search, contact tracing and reviewed hospital records at the health facilities to determine the socio-demographic characteristics, clinical presentation and vaccination status of the cases.

**Results:**

Active case search between November 2015 and January 2016 as well as record review from January 2015 to January 2016 showed that there were 109 suspected and 10 confirmed case patients. We identified 66 cases during the first reported outbreak with a case fatality rate of 6% (4 deaths) while 17 cases were identified 6 weeks later. The epidemic curve indicated a person-to-person transmission.

**Conclusion:**

There had been cases of measles from January 2015 to November 2015 prior to the reported measles outbreak. However there was an unusual increase in the number of measles cases during the measles SIAs in communities where children were missed. Vaccination of all eligible children in the affected wards was carried out. The area council authorities and primary healthcare team need to create awareness on the importance of measles vaccination and ensure that these communities are targeted and covered during subsequent SIAs.

## Introduction

Morbidity and mortality from infectious diseases can be effectively reduced by routine immunization [1, 2]. Globally measles outbreaks continue to be a major public health concern [3, 4]. Measles has been targeted for elimination by the WHO-AFRO region by 2020. In Nigeria, the Expanded Programme on Immunization (EPI) is modelled after World Health Organization (WHO) based guidelines, and works with a national immunization policy which covers free immunization services and provision of potent vaccines, at no cost to populations at risk of vaccine preventable diseases [5]. The EPI schedule in Nigeria expects that infants and children between 12-23 months receive all vaccinations and are considered fully vaccinated if they have received a BCG vaccination against tuberculosis; three doses of penta-valent vaccine to prevent diphtheria, pertussis, tetanus, Hepatitis B, Haemophilus Influenzae type B; at least three doses of polio vaccine; three doses of pneumococal vaccine; two doses of measles vaccination and one dose of yellow fever vaccine [6]. The Nigeria Demographic Health Survey (NDHS) 2013 documents reveals that nationally, only 25% of children age 12-23 months are fully vaccinated; 42% had received measles vaccine, 38% received diphtheria, pertussis, tetanus (DPT) vaccine (still in use at the time of the survey) and 20% did not receive vaccinations at all [6]. The coverage for measles vaccination in North Central zone is 48.1%; this is far from the WHO 90% target for coverage with all vaccines by 2020 [5]. In areas with poor Routine Immunization (RI) coverage, especially in underserved populations, Supplemental Immunization Activities (SIAs), have been adopted as an important part of Nigeria's goal of measles elimination by year 2020 [7]. In Africa the target for interruption of measles virus transmission is to achieve ≥ 95% coverage in every district and community. Also to attain up to 92-94% population immunity by vaccinating at least 95% of children aged 9-59 months with measles vaccine in every settlement, thus providing an opportunity for a second dose of measles vaccination for cases of primary vaccine failure. Properly implemented SIAs serve as an opportunity to minimize outbreaks, strengthen surveillance for measles cases and other infectious vaccine preventable diseases and monitor adverse events following immunization [8, 9]. For diseases targeted for elimination and eradication, the importance of intensified surveillance cannot be overemphasized [4, 7, 10]. Generally measles SIAs require more time and logistics than other types of immunization and involves planning for interventions. This planning involves enumeration of eligible children, micro-planning, vaccine procurement and distribution, cold chain maintenance, training and supervision of qualified personnel, monitoring of adverse events following immunizations (AEFI), injection practices and coverage, waste management, social mobilization and stakeholders meeting and advocacy. These activities are usually achieved by pre-implementation planning, implementation activities and post-implementation assessment. Our objectives in the outbreak investigation were to confirm the outbreak, determine the magnitude and characterize the outbreak in terms of time, place and person. We documented our experiences, which occurred during the implementation of measles SIAs in a semi-urban town in North Central Nigeria.

## Methods

**Study setting:** the setting was in Gwagwalada Area Council of the Federal Capital Territory (FCT) of Nigeria Abuja, which is located in North-Central Nigeria and one of the six area councils of the FCT. Gwagwalada is also the name of the main town in the area council. The Gwagwalada area council is made up of ten wards, namely altogether there are 143 settlements in Gwagwalada area council. The predominant occupation of the people is farming and trading, with majority of the community inhabitants having up to secondary school level education.

**Study population:** Gwagwalada area Council has a total population of 492,800 persons and a target population of 83,776 children less than five years (< 5 years). There are 34 public and 4 private health facilities offering RI services in the Area Council/Local Government Area (LGA). The health facilities are not evenly distributed across the wards in the area council, based on population density for RI [6].

**Implementation activities:** this first phase of the SIAs was conducted in the North-West, North-East and North-Central zones and were conducted nationally in 19 Northern States of Nigeria in November 2015. We conducted health facilities and community based surveys to assess the implementation of the SIAs in Gwagwalada area Council of the FCT Abuja (North-central zone). The SIAs was implemented over a four day period. During the assessment the team discovered an ongoing suspected measles outbreak which had affected 66 children in mainly five settlements in three wards; Angwa Dodo and Dagiri in Central Ward, Angwa Keshi in Kutunku ward, Bargada and Allamu in Paiko ward and those who had presented to the health facilities. Following the suspicion of the outbreak, the Disease Surveillance Notification Office (DSNO) notified the head of health department of the area council, the FCT Primary Healthcare Development agency, the Federal Ministry of Health and other stakeholders. An outbreak investigation team was formed and comprised of disease control officers, DSNO, assistant DSNO and health workers in charge of the health facilities where suspected cases had presented to.

**Case identification:** we conducted community based active case search and included cases reported to the Health Facility (HF) as well as record reviews. Records showed there had been five previous outbreaks in different settlements and wards in the area council over a 10 months period. Names of affected persons in the ongoing outbreak at the time of the SIAs were line listed indicating date of onset of, and dates patients reported in the HF.

We identified cases by using standard Integrated Disease and Surveillance Response (IDSR) case definition for measles, clinical features and recorded the number of cases reported in HFs. We defined a measles outbreak as five cluster cases of measles in a community or a Local government area/ three laboratory confirmed cases from five blood specimens collected in the laboratory. For our case definition, suspected cases were defined as any person in Gwagwalada with onset of fever and body rash between 1st November 2015 and 12th January 2016 with any one of red eyes (conjunctivitis), catarrh or runny nose (coryza) and cough within the three weeks preceding the SIAs. Probable cases were defined as suspected cases with 3 days of rash or known exposure to someone with laboratory-confirmed measles. Confirmed case patients were defined as suspected or probable cases with Koplik spots or positive titer for immunoglobulin (Ig) M antibody.

**Laboratory method:** a total of 16 samples were collected out of the 83 cases (10 from the first 66 cases and 6 from the second 17 cases) and taken to the measles reference laboratory in Maitama, Abuja. Ten of the samples were positive for IgM antibodies, thus confirming a measles outbreak.

**Data processing, data management and analysis:** we adopted descriptive methods for our analysis. Data abstracted from the line-lists was entered using Microsoft Excel 2010 and analyzed using Epi Info 7 Version. We summarized information from record review of suspected measles cases to determine the socio-demographic characteristics, clinical presentation and vaccination status and the measles outbreak investigation summarized using frequencies, means and proportions presented in charts, graphs and tables.

**Ethical considerations:** approval was sought for and gotten from the Department of Primary Healthcare and Public Health Gwagwalada area Council, Abuja FCT. Informed consent was obtained from parents or caregivers of affected children and assent gotten from older children prior to sample collection, confidentiality was assured and maintained for all information collected.

## Results

**Figure 1:**
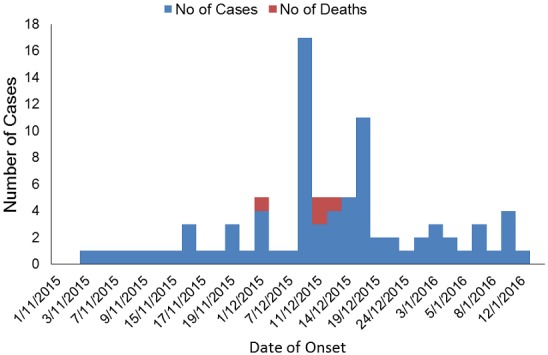
Measles cases and deaths by date of onset of outbreak, Gwagwalada, Abuja-Nigeria, November 2015-January 2016 (n = 83)

A total of 119 suspected and confirmed measles cases based on standard IDSR case definition, were detected out of which 83 suspected cases were identified during the outbreak from 1st November 2015 to 12th January 2016. The first outbreak was from 1st November-24th December 2015 and the second outbreak was from 2nd January to 9th January 2016. A total of 16 (19.2%) of these 83 cases had their blood samples collected for laboratory confirmation out of which altogether 10 (63%) samples tested positive for measles IgM. We identified 66 suspected cases during the first reported outbreak with a case fatality rate of 6% while 17 suspected cases were identified six weeks later with no deaths, the epidemic curve showed a person-to-person transmission (Figure 1).

**Figure 2:**
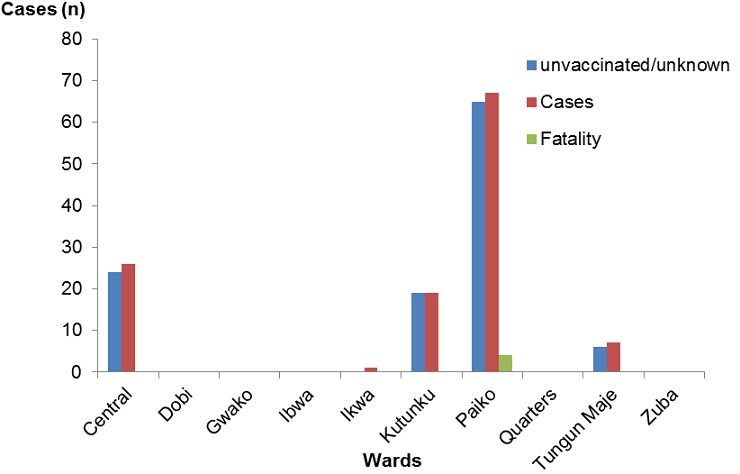
Distribution of measles cases and fatality by vaccination status in wards in Gwagwalada, Abuja-Nigeria, January 2015-January 2016 Nigeria (N = 119)

**Figure 3:**
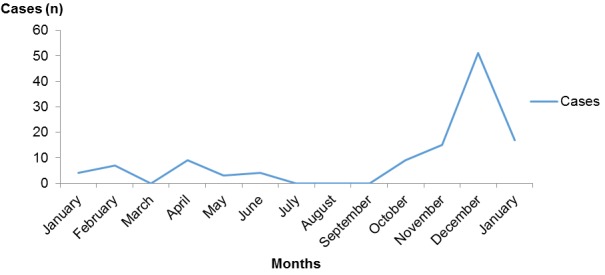
Trend and distribution of suspected and confirmed measles cases in Gwagwalada, Abuja-Nigeria, January 2015- January 2016 (N =119)

Most of the cases during the two reported outbreaks survived the infection with an overall Case Fatality Rate (CFR) of 4.8% (Figure 1). Of the four recorded deaths, two were in children < 5 years (18 months and 3 years), the other two in older children (7 years and 15 years); these were all males children and all from Bargada settlement in Paiko ward. Paiko, Central and Kutunku wards, being wards with highest measles mortality and morbidity (Figure 2). Record review from January to October 2015 showed that there were 36 suspected and five confirmed cases positive with measles IgM. These figures were aggregated from surveillance data collected using measles case based investigation forms. Altogether over a one-year period, there were a total 119 measles cases with cases peaking just before the SIAs, culminating in the outbreak (Figure 3).

The median age of cases was 4 years (range 3 months to 33 years). Over 50% of cases were diagnosed at the health facility and males constituted 53% of cases. More of the cases were unimmunized, partially immunized (one dose), or had unknown immunization history with up to 94% of affected children, having poor vaccination status (Table 1). Four cases of measles were recorded in adults from age range 17-33 years with median age of 22.5 years. The Attack Rate (AR) was 24 per 100,000 population, with age specific AR being 78 per 100,000 population for children less than (<) 5years and 10 per 100,000 population for children/persons 5 years and above.

**Table 1:** Demographic and clinical characteristics of measles cases, Gwagwalada, Abuja, Nigeria November 2015-January 2016 (n = 83)

Characteristics	Frequency (n)	Percentage (%)
**Age Group**		
< 9months	2	2.4
9-60months	54	65.1
>60 months	27	32.5
**Sex**		
Male	44	53.0
Female	39	47.0
**Vaccination status**		
Measles vaccine at 9 months	5	6.0
None	12	14.5
Unknown	66	79.5
**Clinical symptoms**		
Fever	83	100.0
Rash	83	100.0
Cough	83	100.0
Coryza	73	87.9
Conjunctivitis	64	77.1
Diarrhoea	14	33.3
**Outcome**		
Alive	79	95.2
Not alive	4	4.8

Majority of the cases met the case definition criteria for measles infection. Most of the cases were within the 9-60months age group, were male, and up to 94% had not previously received measles vaccine or had unknown vaccination status

**The Supplemental Immunization Activities (SIAs):** the SIAs in the area council was conducted over a four day period. Targeted age groups were children 9-11 months and those 12- 59 months. The findings from the SIAs aggregated call-in data showed that of the children who had never been vaccinated, more children aged 12-59 months had zero dose of measles vaccination, compared to children in the other age groups (Table 2). Of the 104 measles cases who had not been vaccinated in Gwagwalada, 66 (55%) were also of this age group (Table 1). Review of the SIA campaign records showed that 4948 children (6%) aged 12-59 months had not previously received a measles vaccine. The 82,491 children vaccinated were 98% of the target population, with 95% coverage for the measles SIAs, obtained in this community post-SIAs implementation (Table 2).

**Table 2:** Coverage for measles vaccination during northern SIAs, Gwagwalada, Abuja-Nigeria, November 2015 N = 492,800 (total population), n = 83,776 (target population)

	Zero dose 9-11 months	Zero dose 12-59 months	Other dose 9-11 months	Other dose 12-59 months	Vaccine Use	Children vaccinated	Cummulative coverage (%)
Day 1	1,063	1,307	3,633	17,497	24,610	23,500	28
Day2	1,025	1,442	3,027	15,581	22,630	21,075	54
Day 3	527	1,003	2,290	13,759	19,340	17,579	75
Day 4	1,050	1,196	3,239	14,852	21,490	20,337	95

Close to 5000 children in the 12-59 months age range (6%) of children vaccinated had zero dose of measles vaccine, prior to the SIAs. The SIAs was completed with 95% coverage achieved

## Discussion

Our objectives in the outbreak investigation were to confirm the outbreak, determine the magnitude and characterize the outbreak in terms of time, place and person. The confirmed measles outbreak in Gwagwalada occurred during a SIAs campaign which was implemented as a second opportunity for unvaccinated children and eligible children who had not been previously fully immunized after first dose of the measles vaccine to access the vaccine.

Despite availability of a safe, cheap and effective vaccine, measles outbreaks continue to occur in developing countries, with significant morbidity and mortality, especially in developing nations with poor RI coverage including Nigeria [7, 11, 12]. In this outbreak, majority of measles cases had poor vaccination history with over 95% of cases being unvaccinated, thus causing accumulation of susceptible children in the younger age group less than 59 months, who constituted majority of cases, facilitating the person to person transmission of the disease, which was the pattern observed. This is consistent with previously conducted studies which have shown that measles outbreaks, occur more in communities or countries where more children in this at risk age group had been unvaccinated, had incomplete vaccinations or unknown vaccination status [3, 7, 13, 14].

Of the total number of cases in the outbreak, four (4.8%) occurred in persons > 15 years of age. This highlights the fact that adults may be susceptible to measles infections in settings were herd immunity is low. Unvaccinated persons or persons with uncertain immune status may have accumulated over time. This is possible if they were not eligible at the time of the last SIAs, were missed or if campaigns had been few and far between as in the case of this community [8, 12, 15].

Males accounted for more than half (55%) of total measles cases in this setting. Review of previous outbreaks of measles in Nigeria and other developing countries showed that more than 50% of exposed children who developed measles infections were male [3, 13, 14], this was however different from a similar study in Zaria in North Central Nigeria which showed more female cases [16]. In outbreaks in adult populations, similar findings to ours have been documented with evidence that males were up to two times more susceptible to measles than females [17]. The sex of cases however has not been found to be a statistically significant protective or risk factor (determinant) for contracting the infection [5, 13, 14, 16-18].

Though there is strong evidence that during outbreaks more males have a higher susceptibility to viral infectious diseases including measles [15, 17], differences for measles mortality by sex showed that measles mortality was generally higher in females during outbreaks [1, 18, 19]. This was not corroborated in Gwagwalada area Council, as all cases of measles mortality in affected settlements were males.

The 3.4% (overall) and 6% (from the outbreak) CFR for the measles cases and the affected cases in the outbreak seen in this community is consistent with findings in other developing countries. Though as high as 30% during severe outbreaks, CFR for measles infection in Africa is said to range from 3-5%, with rural-urban variations of up to 7-12% in different settings. As the case was in this setting in Gwagwalada, an estimated 50% of mortalities have been documented to occur in children 12-24 months or children less than 5 years, which was the most affected age group in this outbreak [3, 4, 7, 12].

As part of measles elimination across all countries, one of the aims for Africa regional elimination is to reduce measles incidence to < 5 cases per 106 population per year across all the countries [7]. The Attack Rates (AR), including age specific AR show that this community is a high measles burdened community. as many countries in Europe and developing countries in Africa recorded a less than 5 cases per 100,000 incidence of measles based on measles case based surveillance, during an outbreak and following SIAs [8, 13-15].

In high burden communities and countries, the frequency of SIAs is determined by prevailing routine immunization coverage as well as population dynamics and socio-demographics. Measles transmission has been said to be unlikely controlled by a single SIA, but by regular high coverage level SIAs. For Nigeria it has been recommended that SIAs should be scheduled between a 2-3 years interval [9, 11]. Despite following these afore-mentioned recommendations and SIAs modelling strategy and irrespective of the high coverage following the SIAs in Gwagwalada community, six weeks after the November SIAs, another measles outbreak occurred. This observed pattern of increased outbreaks of measles after SIAs campaigns is not uncommon in Nigeria and the situation has been found to be attributable to poor routine immunization coverage and accumulation of missed children in underserved populations in Hard To Reach (HTR) areas [7, 12]. Furthermore sub-optimal conditions in which vaccinations and health care services are delivered have also been implicated in outbreaks occurring after SIAs [2, 5, 20, 21].

These scenarios played out in Gwagwalada, as most of the cases detected during both outbreaks occurred in Bargada and Allamu in Paiko ward, which have terrains with HTR settlements with pastoralist, nomadic families and have poor access to health facilities, health information and health interventions. These HTR settlements alone accounted for 26 (79%) of the cases aged > 59 months, two (50%) of the older cases aged > 15 years, the 66 cases (55%) of morbidity and four (100%) of the mortality amongst the measles cases in Gwagwalada.

Most of the children in these HTR settlements were missed and had sub-optimal vaccination status. This is similar to evidence in certain communities and countries where migration in very mobile populations (nomads, displaced persons), geographical disadvantage due to HTR terrains, in addition to poor access to health services have contributed to measles outbreaks despite high administrative and post-SIA survey coverage [2, 8 ,12, 15, 16, 20, 21].

## Conclusion

The SIAs were continued as scheduled and intensified in affected settlements and wards. Serial walk through assessments were conducted in the community, schools and places of worship, to ensure eligible children were not missed. Effective case management was carried out via a central and accessible designated health post. We carried out community dialogue and advocacy as well as health education and sensitization within the community and to stakeholders. The measles outbreaks in Gwagwalada area Council occurred due to poor routine immunization coverage. Most of the children were unvaccinated or had unknown vaccination history resulting in an accumulation of susceptible children within the area council. There was however an unusual increase in the number of measles cases during the Measles SIAs in communities where children were missed.

Lessons learned from this outbreak investigation are that disease surveillance (measles case based surveillance), disease notification and prompt investigation are important in the control of measles outbreaks. The outbreak may have been avoidable in HTR settlements were children were missed, with proper enumeration and micro-planning in the pre-implementation phase of SIAs especially in the outbreak location. In places with underserved populations and hard to reach areas where peculiar needs exist, supervisory assessment of SIA implementation should be prioritized. These lessons have been highlighted by our experiences shared. The measles SIAs provided these children a second opportunity for measles immunization. We recommended that the area council authority creates awareness on the importance of measles vaccination and ensure that all communities especially those with hard to reach terrain are targeted during subsequent well timed SIAs. Routine immunization should be strengthened by improving availability and accessibility of vaccinations to target population.

### What is known about this topic

- Measles outbreaks occur despite availability of a safe, cheap and effective vaccine;
- When these outbreaks occur, it is with significant morbidity and mortality, especially in developing nations with poor RI coverage;
- Supplemental Immunization Activities (SIAs), have been adopted as an important part of the goal of measles elimination and provide a second opportunity for measles vaccination.

### What this study adds

- Adequate and proper SIAs pre-implementation activities are important for good vaccination coverage, successful implementation of SIAs and subsequent prevention of disease outbreaks;
- Unvaccinated persons and underserved populations (in hard to reach areas with accumulation of susceptible children) may form the focus for an outbreak.

## Competing interests

The authors declare no competing interests.
